# Conformational dynamics of the essential sensor histidine kinase WalK

**DOI:** 10.1107/S2059798317013043

**Published:** 2017-09-27

**Authors:** Yongfei Cai, Mingyang Su, Ashfaq Ahmad, Xiaojie Hu, Jiayan Sang, Lingyuan Kong, Xingqiang Chen, Chen Wang, Jianwei Shuai, Aidong Han

**Affiliations:** a State Key Laboratory for Cellular Stress Biology, School of Life Sciences, Xiamen University, Xiang’an, Xiamen 361102, People’s Republic of China; b Department of Physics, Xiamen University, Xiang’an, Xiamen 361102, People’s Republic of China

**Keywords:** sensor histidine kinase, two-component systems, bacterial signal transduction, helical bending, WalK

## Abstract

Two-component systems (TCSs) are key elements in bacterial signal transduction in response to environmental stresses. TCSs generally consist of sensor histidine kinases (SKs) and their cognate response regulators (RRs). Many SKs exhibit autokinase, phosphoryltransferase and phosphatase activities, which regulate RR activity through a phosphorylation and dephosphorylation cycle. However, how SKs perform different enzymatic activities is poorly understood. Here, several crystal structures of the minimal catalytic region of WalK, an essential SK from *Lactobacillus plantarum* that shares 60% sequence identity with its homologue VicK from *Streptococcus mutans*, are presented. WalK adopts an asymmetrical closed structure in the presence of ATP or ADP, in which one of the CA domains is positioned close to the DHp domain, thus leading both the β- and γ-phosphates of ATP/ADP to form hydrogen bonds to the ∊- but not the δ-nitrogen of the phosphorylatable histidine in the DHp domain. In addition, the DHp domain in the ATP/ADP-bound state has a 25.7° asymmetrical helical bending coordinated with the repositioning of the CA domain; these processes are mutually exclusive and alternate in response to helicity changes that are possibly regulated by upstream signals. In the absence of ATP or ADP, however, WalK adopts a completely symmetric open structure with its DHp domain centred between two outward-reaching CA domains. In summary, these structures of WalK reveal the intrinsic dynamic properties of an SK structure as a molecular basis for multifunctionality.

## Abstract 2

The different conformations of WalK reveal the intrinsic dynamic properties of a sensor histidine kinase structure as a molecular basis for multifunctionality.

## 1. Introduction   

Two-component systems (TCSs) are major signal transduction pathways in bacteria and archaea that play a role in the adaptive response to environmental stresses (Stock *et al.*, 1989 ▸; Mascher *et al.*, 2006 ▸; Krell *et al.*, 2010 ▸). A typical TCS is composed of a sensor histidine kinase (SK) and a cognate response regulator (RR), which are encoded by genes that are often clustered in the genome. While the majority of SKs have multiple domains, all SKs have an invariable core region (∼250 amino acids) consisting of two domains: a dimerization and histidine phosphorylation domain (DHp) and a catalytic ATP-binding domain (CA). Some SKs are able to perform their catalytic activity as full-length SKs *in vitro* in the absence of all other sensor domains (Gutu *et al.*, 2010 ▸).

Many SKs are multifunctional enzymes (Stock *et al.*, 2000 ▸). Modulated by stimulating signals, an SK first performs an autokinase activity and phosphorylates a conserved histidine in its DHp domain. The activated SK then performs a phosphoryltransferase activity to its cognate RR. The phosphorylated RR oligomerizes to activate a variety of cellular functions, one of which is to reprogram prokaryotic cells through transcriptional regulation (Gao & Stock, 2009 ▸). The same SK is often able to dephosphorylate and inactivate the RR.

The current structures of SKs indicate that they may undergo global conformational changes. The structure of the SK HK853 from *Thermotoga maritima* indicates that the DHp domain is sandwiched symmetrically by two CA domains (Marina *et al.*, 2005 ▸). Similarly, the symmetric structures of SKs in *Bacillus subtilis* Spo0B–Spo0F and *T. maritima* ThkA–TrrA complexes have been proposed to represent a phosphoryltransferase state (Varughese *et al.*, 2006 ▸; Yamada *et al.*, 2009 ▸). The CA domains fold close to the DHp domain in the structure of HK853 complexed with its cognate RR468, thus resulting in a distance of approximately 10 Å between the ADP β-phosphorus (βP) and the ∊-nitrogen (∊N) of the phosphorylatable histidine, which in HK853 is interpreted as a phosphatase state (Casino *et al.*, 2009 ▸). Recent structural characterizations of a thermosensor SK, DesK, strongly supports these observations (Trajtenberg *et al.*, 2016 ▸). However, direct connections between these conformations to its specific enzymatic activities still remain uncertain.

In addition to the mobile CA domain, helical bending of the DHp domain has been observed. The crystal structures of DesK indicate that there is a pronounced helical bending in its DHp domain while its CA domain is located in different positions relative to the DHp domain (Albanesi *et al.*, 2009 ▸). Our previous crystal structure of the entire cytoplasmic region of *Streptococcus mutans* VicK also reveals that the asymmetrical positioning of the CA domains is coordinated with a helical bending of the DHp domain (Wang *et al.*, 2013 ▸). Consistently, a DHp domain from the *Escherichia coli* SK CpxA shows that an asymmetric helical bending possibly plays a role in a conformational switch between phosphatase and phosphoryltransferase (Mechaly *et al.*, 2014 ▸). However, the helical bending of the DHp domains of CpxA is not as significant as that observed in VicK.

To further understand the relationship between DHp bending, CA repositioning and ATP binding in autokinase activation, we have used WalK, also known as VicK/YycG, as a model system because of its essentiality in several Gram-positive bacteria, including *Bacillus*, *Streptococcus* and *Staphylococcus* (Wagner *et al.*, 2002 ▸; Senadheera *et al.*, 2005 ▸; Gutu *et al.*, 2010 ▸; Dubrac *et al.*, 2008 ▸). We have recently determined a crystal structure of the entire intracellular domains of VicK from *S. mutans* (smVicK), but it lacked a clear definition of ATP in its binding pocket (Wang *et al.*, 2013 ▸). The structure of the CA domain of *B. subtilis* WalK (bsWalK) has been solved in the presence of ATP but it lacks a DHP domain (Celikel *et al.*, 2012 ▸). Here, we sought to determine the crystal structures of a WalK homologue, lpWalK from *Lactobacillus plantarum*, which shares 60% sequence identity in the DHp and CA domains to our previous solved smVicK structure, in different conformations in the presence of ATP analogues or ADP. We then compared the binding capability of these nucleotides with that of AMP. We finally recapitulated the transitions of the DHp domain using computational simulations. Our data suggest that ATP/ADP binding may affect the dynamics of the WalK conformation in the different enzymatic states.

## 2. Materials and methods   

### 2.1. Protein expression and purification   

A gene fragment encoding lpWalK (amino acids 370–624) was amplified by PCR using *L. plantarum* ATCC 14917 genomic DNA as a template and was ligated into the pET-His expression vector (Mao *et al.*, 2011 ▸). The lpWalK fragment was expressed with an N-terminal His tag in *Escherichia coli* BL21 (DE3) cells induced by 250 µ*M* isopropyl β-d-1-thiogalactopyranoside (IPTG) for 8 h at 25°C. The cells were harvested and resuspended in lysis buffer (50 m*M* Tris–HCl pH 8.0, 500 m*M* NaCl, 10% glycerol, 20 m*M* imidazole, 5 m*M* β-mercaptoethanol) and lysis was completed by sonication. The supernatant was collected after centrifugation at 40 000*g* and was incubated for 1 h at 4°C with 1 ml Ni^2+^–NTA resin (BBI life science) pre-equilibrated with the lysis buffer. After being washed with the lysis buffer, the protein on the beads was eluted with an imidazole gradient of 50–500 m*M*. The best fractions of the protein eluates were concentrated using a Centricon (Millipore) and loaded onto a Superdex 200 size-exclusion chromatography column (GE Healthcare) in a buffer consisting of 20 m*M* Tris–HCl pH 8.0, 100 m*M* NaCl, 5 m*M* β-mercaptoethanol. Peak fractions were collected and concentrated to 15 mg ml^−1^. Initial screening was set up using sitting-drop vapour diffusion with several commercial crystallization kits. The best protein crystals were found in 50 m*M* bis-tris pH 5.6, 1.0 *M* ammonium sulfate, 1% polyethylene glycol 4000. To obtain the ATP-bound state, 4 m*M* AMPPCP and 4 m*M* MgCl_2_ were added to the protein preparation before crystallization. Selenomethionine (SeMet)-labelled WalK was expressed in SeMet-substituted minimal M9 medium and was purified and crystallized as for the native protein (Doublié, 1997 ▸).

### 2.2. Structure determination and refinement   

Crystals were flash-cooled in liquid nitrogen with a cryoprotectant buffer [25%(*v*/*v*) glycerol in mother-liquor solution]. Diffraction data sets were collected with a Quantum 315 detector at station BL17U1 at the Shanghai Synchrotron Radiation Facility (SSRF), Shanghai, People’s Republic of China (Wang *et al.*, 2015 ▸). The data were indexed, processed and scaled with *HKL*-2000 (Otwinowski & Minor, 1997 ▸). The closed lpWalK structures were determined using the molecular-replacement module in *Phaser* with the CA domain of smVicK as a search model (McCoy *et al.*, 2007 ▸). The open lpWalK structure was solved using single-wavelength anomalous dispersion of selenium with the *AutoSol* and *Phaser* programs in *PHENIX* (Adams *et al.*, 2010 ▸). Model building and refinement of these structures were completed using *Coot* and *PHENIX* (Emsley & Cowtan, 2004 ▸; Adams *et al.*, 2010 ▸; Winn *et al.*, 2011 ▸). The statistics of data collection and model refinement are summarized in Table 1 ▸.

### 2.3. Model analysis and graphics   

Model analyses were performed using a variety of programs. The helical bending was calculated using *DynDom* (Taylor *et al.*, 2014 ▸). The buried surface areas were calculated in *StrucTools* (Sanner *et al.*, 1996 ▸). Molecular movies were made in *VMD* and *Chimera* (Humphrey *et al.*, 1996 ▸; Pettersen *et al.*, 2004 ▸). The structural alignments, as well as other graphics, were made in *PyMOL* (Schrödinger) and *Coot* (Emsley & Cowtan, 2004 ▸).

### 2.4. Molecular dynamics   

All MD simulations were performed in an explicit solvent system with constant parameters (NPT; *T* = 310 K, *P* = 101 325 Pa) using *NAMD* 2.8 (Phillips *et al.*, 2005 ▸). The force field was set up by CHARMM27/CMAP (MacKerell *et al.*, 1998 ▸, 2004 ▸). Short-range and nonbonded interactions were calculated using a distance cutoff of 12 Å. The system was neutralized using 200 m*M* NaCl solvated in an 80 Å^3^ box, minimized for 1000 steps and simulated with periodic boundary conditions having full particle-mesh Ewald electro­statics. All MD simulations were analyzed by principal component analysis (PCA) in *Bio*3*D* (Grant *et al.*, 2006 ▸). PCA minimizes the maximal variance of data to fewer dimensions through examining inter-conformer relationships that originate from the Cartesian coordinates of all superimposed structures.

### 2.5. Isothermal titration calorimetry   

Isothermal titration calorimetry experiments were performed using a Malvern MicroCal iTC200 system at 25°C. Here, the lpWalK homologue smVicK was also used because the lpWalK CA domain was not stable. The smVicK gene fragments encoding the DHp/CA domains (amino acids 196–450) and the CA domain (amino acids 271–450) were cloned in the pET-His vector, and the proteins were expressed and purified as described above. All lpWalK and smVicK samples were prepared in the same buffer consisting of 20 m*M* Tris–HCl pH 8.0, 5 m*M* MgCl_2_. The sample cell was filled with 50 µ*M* VicK and titrated with the ligands AMPPNP, ADP or AMP at concentrations that were empirically determined to have the best signal-to-noise ratio. The concentrations of AMPPNP and ADP were set to 3 m*M* and the concentration of AMP was set to 6 m*M*. The delay time was 60 s for the first titration of 0.4 µl of ligand. The rest of the titrations were completed with 2 µl of ligand per each injection for 20 intervals of 120 s. The stirring speed was 1000 rev min^−1^. The reference power was 5 µcal s^−1^. The data were fitted with a fixed 1:1 binding model in the *Microcal Analysis Launcher* software package. More than 200 iterations were used to reduce the χ^2^ until all values remained constant. The final *K*_d_, Δ*H* and Δ*S* values after the fitting are displayed on the plot.

## 3. Results   

### 3.1. The structure of WalK in the closed state   

**Figure 1:**
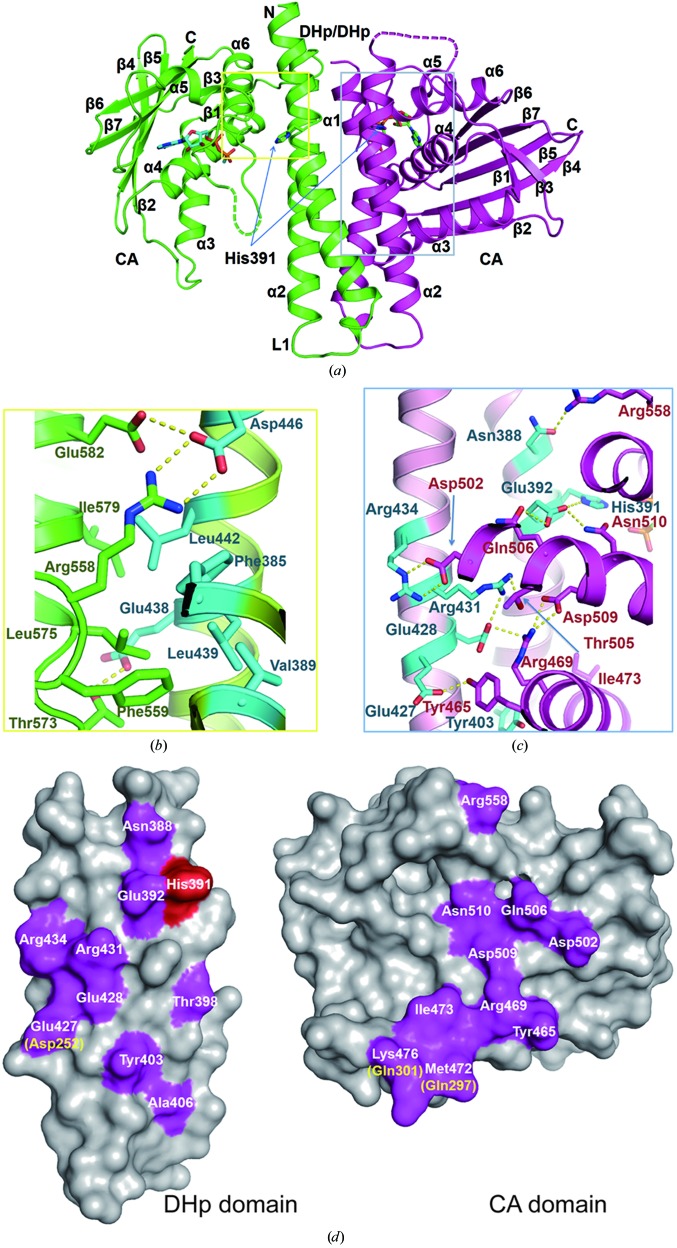
The crystal structure of WalK in the closed state. (*a*) The asymmetrical structure of closed lpWalK bound by the ATP analogue AMPPCP shown as sticks and spheres. The closed monomer is coloured purple and the open monomer is coloured green. (*b*) The interface between the CA and DHp domains of the open monomer is indicated by a yellow box in (*a*). Key interacting residues from the DHp domain are shown as cyan sticks and those from the CA domain are shown as green sticks. (*c*) The interface between the CA and DHp domains of the closed monomer is indicated by a blue box in (*a*). Key interacting residues from the DHp domain are shown as cyan sticks and those from the CA domain are shown in purple. Hydrogen bonds are highlighted by yellow dashed lines. (*d*) The conservation of key residues in the closed DHp and CA interface between lpWalK and smVicK. The conserved residues in purple are highlighted and labelled based on lpWalK, and the nonconserved residues in smVicK are indicated in yellow in parentheses. His391 is coloured red.

The catalytic region of lpWalK contains one DHp domain (amino acids 370–450) and one CA domain (amino acids 450–624). To obtain ATP-bound states, we crystallized the lpWalK fragment in the presence of the nonhydrolyzable ATP analogues AMPPCP and AMPPNP. Surprisingly, both structures resemble the smVicK catalytic region (amino aicds 196–450; Fig. 1*a* and Supplementary Fig. S1*a*). The γ-phosphate in the lpWalK–AMPPNP complex could not be defined, probably because of hydrolysis. However, the overall structure showed the same conformation as lpWalK co-crystallized with AMPPCP (Supplementary Fig. S1*b*). Indeed, we also observed the same conformation for lpWalK that was crystallized in the presence of ADP (Table 1 ▸ and Supplementary Fig. S1*b*).

The overall conformations of these nucleotide-bound lpWalK structures represent an asymmetric closed state with one CA domain positioned close to the DHp domain, while the other CA domain reaches out, forming an open conformation (Fig. 1 ▸ *b*). The CA and DHp interactions are *in cis* rather than *in trans*. Residues Arg558 and Phe559 of the F box form a small interface between the DHp and open CA domains through hydrogen bonds and hydrophobic interactions, as observed in smVicK. These residues, together with the other interacting residues, including Thr573, Leu575, Ile579 and Glu582 from the CA domain and Phe385, Leu439, Leu442, Asp446 and Glu438 from the DHp domain, are highly conserved in WalK homologues. The buried surface area is as small as 682.8 Å^2^, but this interface appears to be further stabilized by a hydrogen-bond network consisting of Asp446, Arg558 and Glu582, probably as a result of subtle conformational changes in the F and G2 boxes induced by nucleotide binding.

The CA domain in the closed conformation makes a more extensive interface with the DHp domain (Fig. 1 ▸ *c*). The conserved residues His391 and Glu392 in α1 form the first hydrogen-bond network with Gln506 and Asn510 in α4. Gln506 interacts with Glu392 through a hydrogen bond that is essential for His391 phosphorylation, as has been demonstrated in the chimeric EnvZ (Casino *et al.*, 2014 ▸). Residues Glu427/428 and Arg431/434 in α2 form the second hydrogen-bond network with Tyr465 and Arg469 in α3 and Asp502/509 in α4. Consistently, mutation of these residues in smVicK (Asp326/Gln330) significantly disrupts the autokinase activity (Wang *et al.*, 2013 ▸). Arg558 of the F-box also contributes to this interface through a hydrogen bond to Asn388 in the DHp domain. Several residues, including Thr398, Tyr403 and Ala406 in α1 and Met472, Ile473 and the aliphatic side chain of Lys476 in α3, form a small hydrophobic interface (Supplementary Fig. S2*a*). These key residues in the closed DHp and CA interface are largely conserved between lpWalK and smVicK (Fig. 1 ▸ *d*). Although Glu427 in lpWalK is substituted by Asp252 in smVicK, the hydrogen bond formed by the side chain to Tyr465 is preserved. Similarly, Met472 inlpWalK is substituted by Gln297 in smVicK, but the hydrophobic interaction formed between their side chains and Tyr403 is also preserved. Interestingly, the interaction between Lys476 and Ala406 in lpWalK is not found in smVicK as Lys476 is substituted by Gln301. Together, our data indicate that WalK adopts a closed structure in the presence of ATP analogues or ADP, which is characterized by the asymmetric positioning of the two CA domains; only one monomer of the dimer switches to a closed conformation while the other one remains open. The structures of the two CA domains are nearly identical (r.m.s.d. of 0.36–0.54 Å for 120 C^α^ atoms), except for a 15.3° rotation of α3 toward to the DHp domain in addition to the flexible loops L3 and L5 and the G1 box (Supplementary Fig. S2*b*). The rotation of α3 avoids stereo clashes with the DHp domain when forming the closed conformation.

### 3.2. Nucleotide-binding pocket and histidine   

**Figure 2:**
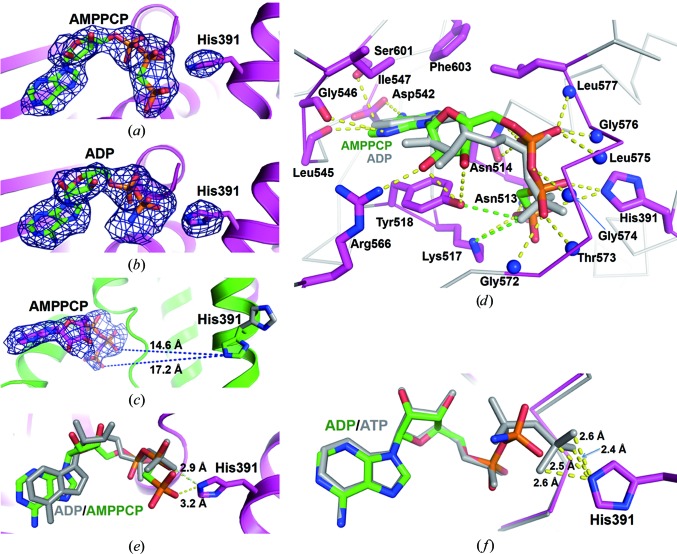
Nucleotide binding in the active site of closed WalK. (*a*, *b*, *c*) Electron-density maps of bound nucleotides and His391. The simulated-annealing OMIT map is contoured at 2σ. An alternative rotamer conformation of His391 in the open chain is shown as grey sticks. Long distances between ∊N and βP/γP are highlighted by blue dashed lines. (*d*) Detailed interaction of AMPPCP and ADP in the active pocket of the CA domain. Key interacting residues are shown as sticks. Hydrogen bonds formed to AMPPCP are highlighted by yellow dashed lines and hydrogen bonds formed to βP in ADP are highlighted by green dashed lines. The backbone amines are shown as balls. (*e*) Aligned AMPPCP and ADP in the active pocket of the CA domain. AMPPCP is coloured green. ADP is coloured grey. The hydrogen bonds from AMPPCP/ADP to His391 ∊N are highlighted and labelled with the bond distances. (*f*) Modelled ATP in the active pocket of the CA domain. ATP was modelled into its pocket by alignment of lpWalK with the bsWalK–ATP complex. ATP is coloured grey. ADP from the lpWalK–ADP complex is coloured green. The possible hydrogen bonds between ATP γP and His391 ∊N are highlighted and their bond distances are also labelled.

In the closed lpWalK state, the ATP/ADP ligands are clearly defined (Figs. 2*a*, 2*b* and 2*c* ▸). The adenine plane of ADP is sandwiched by Tyr518 from the N box on one side and by Ile547 from the G1 box and Phe603 from β7 on the other, and is further fixed by a hydrogen bond between Asp542 and the amine at adenine C6. The ribose plane is orientated by two hydrogen bonds from the 2′ and 3′ hydroxyl groups to Tyr518 of the N box and Arg566 between the F and G2 boxes (Fig. 2 ▸ *d*).

The ATP phosphates are also involved in extensive interactions with lpWalK (Fig. 2 ▸ *d*). The α-phosphate (αP) forms hydrogen bonds to the amine of Asn514 and the main chain of residues 574–577 from the G2 box. The β-phosphate (βP) forms hydrogen bonds to Gly572 and Thr573 from the G2 box. Lys517 and Asn513 from α4 can both connect to the γ-phosphate (γP) through hydrogen bonds, while Lys517 and Tyr518 both form hydrogen bonds to βP in ADP-bound lpWalK (Fig.  ▸ *d*), consistent with our previous finding that the mutation of homologous residues in VicK (Lys341A/Tyr342A) clearly reduces the autokinase activity (Wang *et al.*, 2013 ▸). Remarkably, His391 ∊N makes two hydrogen bonds to both the βP and γP of AMPPCP (Fig. 2 ▸ *e*). A tight hydrogen-bond network can be postulated between His391 ∊N and ATP γP, as indicated by alignment of the closed lpWalK state with the bsWalK CA structure bound by ATP (Fig. 2 ▸ *f*). In the open conformation, however, these interaction partners are approximately 15–17 Å away, whereas His391 has two rotameric conformations that are fixed through its interaction with AMPPCP or ADP in the closed conformations (Fig. 2 ▸ *c*). Together, our results suggest that ATP/ADP makes extensive interactions with the CA domain as well as with the phosphoryl­atable histidine in the closed conformation.

A magnesium ion was not identified in the ATP/ADP-binding pocket of the closed lpWalK structures. Our alignment of the closed CA domain with the bsWalK structure, where Mg^2+^ is present, showed that the structures are nearly identical (r.m.s.d. of 0.69 Å for 112 C^α^ atoms; Supplementary Fig. S2*c*). Moreover, both nucleotides aligned except for the γP positions, which were 3.3 Å apart (Supplementary Fig. S2*d*). Two coordinations by the hydroxyl groups of βP and γP are lost in our lpWalK structure bound to AMPPCP, which makes lpWalK unable to chelate Mg^2+^ stably.

We then measured the binding affinities of AMPPNP, ADP and AMP for the WalK proteins lpWalK and smVicK using isothermal titration calorimetry (ITC) experiments (Table 2 ▸ and Supplementary Fig. S3). The overall binding affinities of these nucleotides to WalK were in the high-micromolar to millimolar range. ADP and AMP were generally weaker binders than ATP, which was in the *K*_d_ range 300–500 µ*M*.

### 3.3. The structure of WalK in the open state   

**Figure 3:**
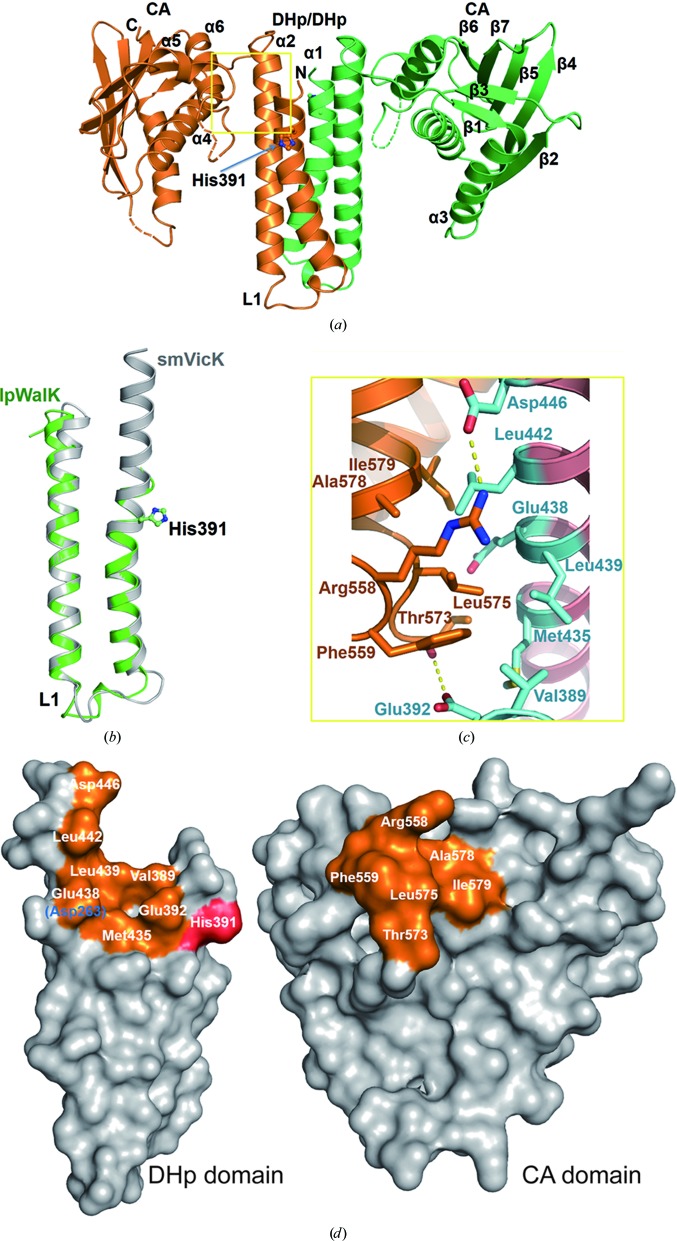
The crystal structure of WalK in the open state. (*a*) The symmetric structure of open lpWalK in the absence of nucleotides. The two chains are coloured gold and dark green. (*b*) Structural similarity of the DHp domain to the inactive chain of smVicK is coloured in grey. The phosphorylatable histidine His391 is shown as sticks. (*c*) The interface between the DHp and CA domains is indicated by a yellow box in (*a*). Key residues are shown as sticks, with their hydrogen bonds in yellow dashed lines. Residues from the DHp domain are coloured cyan. (*d*) The conservation of key residues in the open DHp and CA interface between lpWalK and smVicK. The conserved residues in gold are highlighted and labelled based on lpWalK, and the nonconserved residues in smVicK are indicated in blue in parentheses. His391 is coloured red.

We also determined the crystal structure of the catalytic region of lpWalK at 3.2 Å resolution in the absence of ATP/ADP. Unlike the closed lpWalK described above, the overall structure shows that the two CA domains symmetrically centre on the four-helical bundle of the DHp homodimer (Fig. 3 ▸ *a*). The CA domains reach out, causing the ATP-binding pocket to be positioned further away from the phosphorylat­able histidine His391 in the DHp domain; we therefore defined this structure as representing the open state of WalK.

The structures of both CA domains are similar to that of its close homologue *S. mutans* VicK (smVicK; r.m.s.d. of 0.85 Å for 116 C^α^ atoms; Supplementary Fig. S4*a*; Wang *et al.*, 2013 ▸). It is also similar to that of *B. subtilis* WalK (bsWalK; r.m.s.d. of 0.83 Å for 107 C^α^ atoms; Celikel *et al.*, 2012 ▸). The major differences were the positional shifts, by up to 4.6 Å, of the loops and α3. Interestingly, the ATP pocket covering the loops is only partially ordered in all three structures, even though the bsWalK structure is bound to ATP. The lpWalK DHp domain forms a symmetric dimer, and the helices are both straight and well aligned with the inactive chain of smVicK (r.m.s.d. of 0.54 Å for all C^α^ atoms; Fig. 3 ▸ *b*).

A small interface is formed between residues Arg558, Phe559, Thr573 and Leu575 of the CA domain and Val389, Glu392, Met435, Leu439 and Asp446 of the DHp domain (Fig. 3 ▸ *c*). The key residues in the open DHp and CA interface between lpWalK and smVicK are completely conserved (Fig. 3 ▸ *d*). Although Glu438 is substituted by Asp263 in smVicK, the hydrophobic interaction is preserved as both have similar side chains. The buried surface area is barely 689.5 Å^2^, thus suggesting that this interface is not strong. Moreover, the key residues in the CA domain are located in the lid-covering loop of the ATP-binding pocket, including the F and G2 boxes. Indeed, a global alignment of lpWalK with the open chain of smVicK indicated an ∼4° rotation of the CA domain relative to the DHp (Supplementary Fig. S4*b*). A dramatic rotation (∼46.1°) of the lpWalK CA domain was observed when it was aligned with *T. maritima* HK853 in the open state (Supplementary Fig. S4*c*).

### 3.4. Asymmetrical helical bending in the DHp domain   

**Figure 4:**
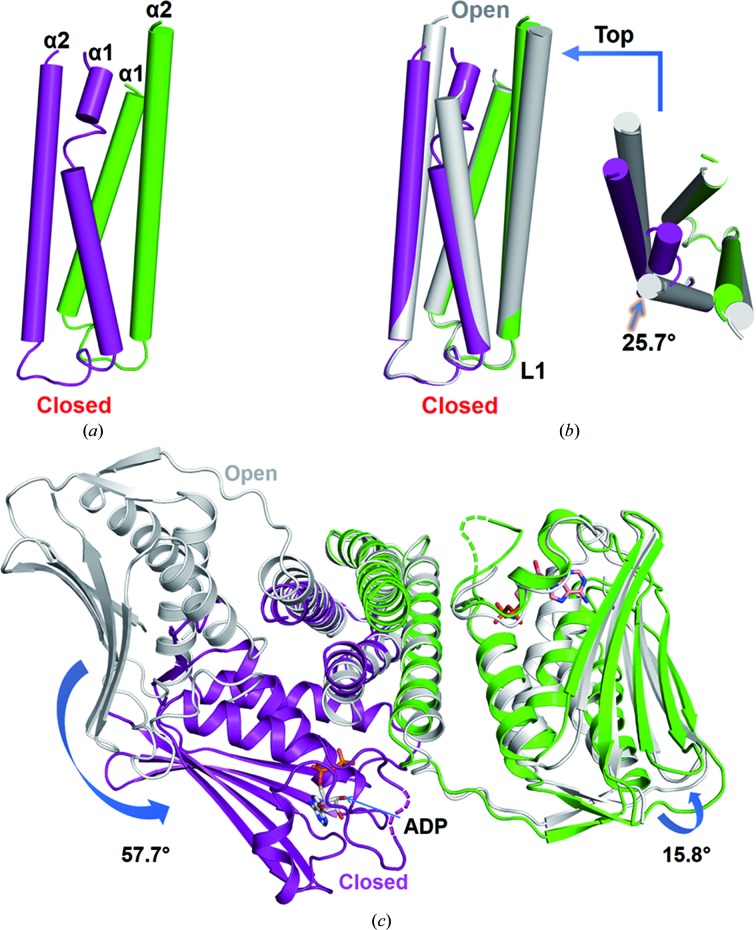
Coordinated DHp helical bending and CA positioning during the open-state to closed-state transition. (*a*) Asymmetrical structure of the DHp domain of closed lpWalK. The bent monomer is coloured purple and the straight monomer is coloured green. (*b*) Alignment of the DHp domain of closed lpWalK with that of open lpWalK. The alignment was performed using the invariable bottom region (amino acids 388–405). A top view of this alignment is shown on the right. The bending direction and degree are marked with an arrow. (*c*) Global structural alignment of closed and open lpWalK. These two structures are aligned and coloured as in (*b*). The rotation of the CA domain is indicated with an arrow. AMPPNP in the CA domains is shown in sticks.

The homodimeric DHp domain in the closed lpWalK structure is asymmetric (Fig. 4 ▸ *a*). The monomer connecting the open CA domain adopts two straight helices, whereas the other monomer α1 bends towards the dimeric centre. When compared with their positions in open lpWalK, both DHp domains are well aligned except for the bent α1 (r.m.s.d. of 0.37 Å for 97 C^α^ atoms; Fig. 4 ▸ *b*). The bending of the α1 N-terminal tail is 25.7° at Pro396. Coordinately, the closed CA domain has a rotation of 57.7° and 9.1 Å difference along the DHp domain, while the open CA domain is positioned with a 15.8° difference (Fig. 4 ▸ *c* and Supplementary Movie S1). The asymmetrical structure of the DHp domain is comparable to that of smVicK, with nearly the same bending angle (r.m.s.d. of 0.78 Å for 59 C^α^ atoms). In the light of recent structural evidence from the SKs CpxA and DesK (Mechaly *et al.*, 2014 ▸; Trajtenberg *et al.*, 2016 ▸), the DHp domain with symmetric straight helices and asymmetric helical bending, in coordination with the CA-domain positioning, may represent two distinct conformations of the WalK family.

### 3.5. The dynamics of the DHp domain   

**Figure 5:**
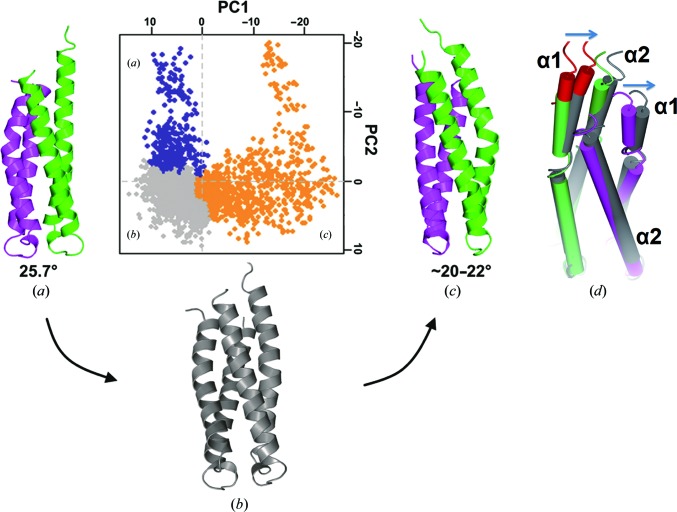
Dependence of the helical bending switch in the DHp dimer. (*a*, *b*, *c*) MD simulation of the DHp homodimer from the closed state of lpWalK for 40 ns. The original state (*a*) and a simulated state at ∼3 ns (*c*) of the DHp dimer are coloured purple and forest green. An intermediate state (*b*) is coloured grey. Helical bending angles are given below. PCA analysis of the MD simulation is shown for the first two PC planes. Blue indicates an assembly close to the state in (*a*), yellow for the state in (*c*) and grey for the state in (*b*). (*d*) Helical bending switch of an engineered DHp homodimer. The DHp dimer in state (*c*) is coloured forest green, and purple was used for rebuilding. A simulated state at 15 ns, coloured grey, was aligned with state (*c*). A rebuilt helical region (amino acids 372–382) taken from the opposite α1 is coloured red. The α1 back-shifting is highlighted with arrows.

To further understand the dynamics of the asymmetric helical bending in the DHp domain, we performed molecular-dynamics (MD) simulations. We observed that two straight α1 helices of the DHp domain from the open lpWalK were randomly bent by 16.7–26.8° during 50 ns of simulation, indicating that the bending is an intrinsic property of the α1 helices (Supplementary Movie S2). In contrast, the DHp domain from the closed lpWalK structure behaved completely differently (Supplementary Movie S3). The straight α1 helix became bent towards the central axis of the DHp domain, while the bent α1 helix retracted and became straight, thus resulting in switched α1 helical bending at ∼3 ns (Figs. 5*a*, 5*b* and 5*c* ▸). The simulated α1 bending angle was ∼20–22° at Pro396, which was slightly smaller than the actual bending observed in the crystal structure. More importantly, only one α1 in the DHp homodimer could bend, thus supporting the sequential bending hypothesis that we proposed previously (Wang *et al.*, 2013 ▸). Principal component analysis (PCA) suggested that such a transition passes through an intermediate state in which both α1 helices bend at small angles (Fig. 5 ▸ *b*).

Surprisingly, the DHp domain retained a bent α1 state (Fig. 5 ▸ *c*) and could not switch back to a straight conformation during the rest of the 35 ns of simulation. We reasoned that the resultant DHp structure might have unequal lengths of the α1 helices. To test this possibility, we simulated the DHp domain after removing the extra helical region (amino acids 372–383) to make the α1 termini of equal lengths. However, this DHp did not exhibit any switches during 20 ns of simulation (Supplementary Fig. S5). We then shifted the helical region (amino acids 375–383) from the bent α1 to the straightened α1 by using the simulated DHp structure in Fig. 5 ▸(*c*) and continued the simulation under the same conditions. We observed that α1 successfully switched back in 15 ns (Fig. 5 ▸ *d*, Supplementary Movie S4). Finally, we tested whether the helical property of the extra helical region was required for this function. We rebuilt the helical region as a random coil during a 40 ns simulation. Although the random coil is positioned freely, the connected α1 did not bend at all (Supplementary Movie S5).

Together, these MD studies revealed that the helical bending of the two α1 N-termini at the kink-residue proline is an intrinsic property of the homodimeric DHp domain, and the length and helicity of the α1 helix determine its helical bending capability. The α1 with a longer N-terminal helix and higher helicity appears to promote a favourable bending towards the central axis of the dimeric DHp domain. Moreover, the two α1 termini exhibit mutually exclusive bending and are able to switch sequentially.

## 4. Discussion   

The ability of one SK to have both phosphorylation and dephosphor­yl­ation enzymatic activities is believed to rely on the flexibility of its conformation, such as that of DesK, HK853 and CpxA, as demonstrated in several structural studies (Albanesi *et al.*, 2009 ▸; Casino *et al.*, 2014 ▸; Mechaly *et al.*, 2014 ▸; Trajtenberg *et al.*, 2016 ▸). A conformationally dynamic nature has also been implicated by computational simulations (Dago *et al.*, 2012 ▸). Here, we report the conformational dynamics of an essential SK, lpWalK, an smVicK homologue from *Lactobacillus*.

### 4.1. The closed conformation of WalK with ATP/ADP bound   

LpWalK adopts a closed conformation in the presence of ATP analogues and ADP, but not in the presence of AMP in our crystallization condition (Fig. 1 ▸). The crystal packing of lpWalK in the closed state was completely different from that in the open state (Table 1 ▸). Several thiazolidione WalK inhibitors, which are predicted to bind to the WalK active pocket (Huang *et al.*, 2012 ▸), did not induce lpWalK to crystallize under the same conditions as used for the lpWalK fragment. Importantly, selective crystallization of one specific conformation of target molecules often occurs. Indeed, in drops containing Mg^2+^ and ATP analogues a small portion of lpWalK crystals, which had the same morphology as those without ATP/ADP, were found to still be in the open state (data not shown). In addition, the γP of ATP was visible when lpWalK was co-crystallized with AMPPCP but not AMPPNP. Interestingly, although ATP is chemically unstable, its γP has been observed in several SK structures, including the bsWalK CA domain and CpxA (Celikel *et al.*, 2012 ▸; Mechaly *et al.*, 2014 ▸).

The closed WalK adopts an asymmetric conformation (Fig. 1 ▸). Only one monomer folds into a closed state through an ∼58° rotation, while the other monomer remains open. The closed monomer has an extensive hydrogen-bond network and hydrophobic interactions between the DHp and CA domains (Fig. 1 ▸ *c* and Supplementary Fig. S2*a*). The open monomer of lpWalK has a small interface between the CA and DHp domains, which is also found in the open lpWalK structure (Figs. 1 ▸ *b* and 3 ▸ *c*). However, it is completely lost in the closed monomer of the closed lpWalK, whereas Arg558 switches its hydrogen bonds from Asp446 in α2 to Asn388 in α1 (Fig. 1 ▸). Such a conformational change also cooperates with the unfolding of the C-terminal α2 helix (amino acids 442–447) of the DHp domain, which has been accurately predicted by a co-evolutional analysis using a direct coupling algorithm (DCA; Fig. 4 ▸ *c*, Supplementary Movie S1; Dago *et al.*, 2012 ▸). Consistently, the corresponding residue Arg382, which is important for autokinase activity in smVicK, also makes a similar switch (Wang *et al.*, 2013 ▸).

ATP/ADP fits into the active site of the CA domain through extensive interactions involving the N box and the G1 and G2 loops (Marina *et al.*, 2001 ▸; Casino *et al.*, 2014 ▸). We have found that ATP/ADP interacts with His391 in the DHp domain through hydrogen bonds in the closed lpWalK (Fig. 2 ▸). Asn510, which is one of two signature residues in the N box, may form a hydrogen bond to Glu392 but not to His391 of the DHp domain (Fig. 1 ▸). Consistently, the corresponding residue Asn343 in EnvZ makes similar contacts, and mutation of this residue disrupts EnvZ autokinase activity without disrupting its ATP-binding affinity (Hsing *et al.*, 1998 ▸; Casino *et al.*, 2014 ▸). Another signature residue, Asn514 in the N box, creates two hydrogen bonds to αP in lpWalK (Fig. 2 ▸ *d*), whereas in the chimeric EnvZ the corresponding residue Asn347 interacts with an Mg^2+^ ion (Casino *et al.*, 2014 ▸). Intriguingly, smVicK with double Ala mutations of these two Asn residues still has observable autokinase activity (Wang *et al.*, 2013 ▸). Instead, Gln506 adjacent to the N box hydrogen-bonds to Glu392 only in the closed lpWalK state (Fig. 1 ▸ *c*), and mutation of this residue disrupts the autokinase activity of smVicK (Wang *et al.*, 2013 ▸). Consistently, the Gln506–Glu392 contact ranks highest among the pairs identified from the DCA analysis (Dago *et al.*, 2012 ▸). Taken to­gether, we propose in lpWalK that Glu392 neighbouring the phosphorylatable His391 acts as a general base, which is further enhanced by two polar residues, Gln506 and Asn510. At the same time, the Mg^2+^ ion chelated by Asn514 and ATP catalyzes γP breakdown.

### 4.2. ATP and ADP bind WalK with weak affinity   

ATP and ADP bind lpWalK/smVicK with different affinities to AMP (Table 2 ▸). ATP is able to bind smVicK or lpWalK at 300–500 µ*M*, which is similar to the binding of CheA but is ∼60-fold weaker than the binding of HK853 (Casino *et al.*, 2014 ▸; Tawa & Stewart, 1994 ▸; Surette *et al.*, 1996 ▸; Stewart *et al.*, 1998 ▸). ADP binds to smVicK at 700 µ*M*, which is eightfold weaker than binding of the *E. coli* PhoQ cytoplasmic region (Yeo *et al.*, 2012 ▸). AMP has extremely weak binding abilities. In agreement with these results, a recent report has indicated that ADP rather than AMP is an efficient VicK kinase inhibitor (Wilke *et al.*, 2015 ▸). Interestingly, all nucleotides bind the CA domain better than the entire catalytic region of smVicK, thus suggesting possible steric hindrance of the DHp domain towards the CA active pocket. Similarly, full-length CheA binds ATP with an ∼20-fold weaker affinity than its ATP-binding domain P4 (Bilwes *et al.*, 2001 ▸). Unfortunately, because the CA domain of lpWalK was not sufficiently stable in our assay, we were unable to verify this observation in lpWalK.

The competitive binding of ATP/ADP may also be regulated by their local cellular concentrations. The concentration of ATP is 2–10 m*M*, which is threefold to 30-fold higher than that of ADP in *E. coli* (Buckstein *et al.*, 2008 ▸; Bennett *et al.*, 2009 ▸). In contrast, the WalK concentration may be very low in Gram-positive bacteria. For example, there are only 920 VicK molecules (at ∼1.5 µ*M*) in one *S. pneumoniae* cell (Wayne *et al.*, 2010 ▸). Therefore, it is plausible to predict that most WalK is likely to remain in the ATP-bound and not the ADP-bound state (Wang *et al.*, 2012 ▸).

### 4.3. The intrinsic dynamics of DHp may be central to its enzymatic complexities   

The DHp domain is intrinsically dynamic. The DHp domain is sandwiched between two CA domains. In the open WalK structure the DHp is completely symmetric, with two straight α1 helices, whereas in the closed WalK state it is asymmetric, as characterized by helical bending in one α1 but not the other (Fig. 4 ▸). A series of MD simulations suggested that the DHp domain with equal-length α1 helices from the open WalK structure behaves with a random bending mode in a 50 ns simulation (Supplementary Movie S2). In contrast, the two α1 helices of the DHp domain from the closed WalK structure completed the first bending switch in ∼3 ns (Figs. 5 ▸ *a*, 5 ▸ *b* and 5 ▸ *c*). Continuous simulation made the α1 helix switch back only when α1 contained an extended helix (Fig. 5 ▸ *d*). These observations support a helix–coil transitional switch mechanism of the N-terminal α1 helices during autokinase activation (Wang *et al.*, 2012 ▸).

The N-terminal α1 helices are also essential for phosphatase activity. The top of the DHp domain containing the α1 helices and the C-terminal α2 regions is very sensitive to mutation because most phosphatase-disruptive mutations are located in these regions (Hsing *et al.*, 1998 ▸). However, the hinge residues threonine and proline, but not the valine and serine residues at the top, are important for phosphatase activity (Willett & Kirby, 2012 ▸; Wang *et al.*, 2013 ▸). However, HisKA_3 family SKs, such as NarX, may have different dynamics from these HisKA SKs because sensitive mutations are located on the bottom of the DHp domains (Huynh *et al.*, 2013 ▸).

SK activities are largely modulated by upstream signals *in vivo*. Some SKs are directly connected to sensor domains and activated by their cognate ligands. A HAMP domain (**h**istidine kinases, **a**denylate cyclases, **m**ethyl accepting proteins and **p**hosphatases) connects a transmembrane domain and allows the transmission of a variety of extracellular stimuli to downstream catalytic activities in many SKs (Dunin-Horkawicz & Lupas, 2010 ▸). The Af1503 HAMP domain is able to control the catalytic activity when it is engineered to the output domains of adenylyl cyclase, chemotaxis receptor and EnvZ (Hulko *et al.*, 2006 ▸; Ferris *et al.*, 2012 ▸). A unique HAMP solution structure has suggested a rotatory potential of its four-helical bundle (Hulko *et al.*, 2006 ▸). Surprisingly, its derivatives with mutation of the key residue Ala291 respond differently to amino acids as stimuli, but adopt essentially the same structure (Ferris *et al.*, 2012 ▸). One possible explanation is that the upstream domains may directly or indirectly modulate the DHp dynamics in response to stimuli.

In summary, these observations suggest that the four-helical bundle of the DHp domain may have internally coordinated motions, which cooperate with the CA-domain repositioning and produce different conformations of WalK. ATP/ADP binding may modulate the dynamics of the CA domain. The dynamic properties of the DHp domain are likely to be essential for the enzymatic activities of WalK, which are largely modulated by upstream functional domains in response to a variety of stimulatory signals.

## Supplementary Material

PDB reference: *Lactobacillus plantarum* WalK, 4u7n

PDB reference: bound to AMPPNP, 4u7o

PDB reference: bound to ADP, 4zki

PDB reference: bound to AMPPCP, 5c93

Supplementary Movie S1.. DOI: 10.1107/S2059798317013043/di5014sup2.mov

Supplementary Movie S2.. DOI: 10.1107/S2059798317013043/di5014sup3.mov

Supplementary Movie S3.. DOI: 10.1107/S2059798317013043/di5014sup4.mov

Supplementary Movie S4.. DOI: 10.1107/S2059798317013043/di5014sup5.mov

Supplementary Movie S5.. DOI: 10.1107/S2059798317013043/di5014sup6.mov

Supplementary Figures and Supplementary Movie captions.. DOI: 10.1107/S2059798317013043/di5014sup1.pdf

## Figures and Tables

**Table 1:** Data-collection and refinement statistics for the crystal structures of lpWalK Values in parentheses are for the highest resolution shell.

	Closed structures			
	+ AMPPCP	+ AMPPNP	+ ADP	Open structure†
Data collection				
Space group	*P*2_1_2_1_2_1_	*P*2_1_2_1_2_1_	*P*2_1_2_1_2_1_	*P*3_1_
Unit-cell parameters				
*a* (Å)	54.65	54.53	53.499	91.767
*b* (Å)	97.75	98.39	96.034	91.767
*c* (Å)	117.34	98.39	119.351	97.717
α = β (°)	90.00	90.00	90.00	90.00
γ (°)	90.00	90.00	90.00	120.00
Wavelength (Å)	0.97915	0.97924	0.97853	0.95370
Resolution range (Å)	50–2.5 (2.54–2.50)	25–2.4 (2.44–2.40)	50–3.4 (3.46–3.40)	25–3.0 (3.05–3.00)
Completeness (%)	97.9 (99.9)	99.5 (100)	99.8 (98.6)	99.7 (99.6)
*R*_merge_ ‡ (%)	5.9 (68.6)	10.0 (71.9)	14.6 (56.7)	7.1 (91.9)
Multiplicity	4.7 (4.8)	6.4 (7.3)	5.6 (3.9)	5.7 (5.4)
*I*/σ(*I*)§	25.358 (2.04)	38.163 (4.16)	10.000 (1.77)	37.594 (2.00)
Refinement				
Resolution range (Å)	37.01–2.52	14–2.40	50–3.45	25–3.20
No. of reflections used	21206	25012	8881	29907
*R*_work_ ¶/*R*_free_ †† (%)	21.8/27.4	19.82/23.86	20.14/22.20	25.09/28.48
No. of atoms				
Protein	3666	3628	3555	3394
Ligand	82	54	27	—
Water	6	34	—	—
Average *B* value (Å^2^)	74	63.0	74	159
R.m.s.d.				
Bonds (Å)	0.009	0.011	0.011	0.008
Angles (°)	1.271	1.451	1.437	1.170
Ramachandran plot				
Most favoured (%)	97.1	98.19	95.56	94.89
Allowed (%)	2.9	1.81	4.44	5.11
Disallowed (%)	0	0	0	0
PDB code	5c93	4u7o	4zki	4u7n

† The open structure was determined using selenomethionine-substituted protein and single-wavelength anomalous dispersion (SAD).

‡ *R*_merge_ = 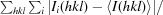 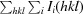, where *I_i_*(*hkl*) is the observed intensity and 〈*I*(*hkl*)〉 is the statistically weighted average intensity of multiple observations of symmetry-related reflections.

§ *I*/σ(*I*) is the ratio of the mean intensity to the mean standard deviation of intensity.

¶ *R*_work_ = 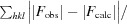 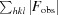, where *F*_obs_ and *F*_calc_ are the observed and calculated structure factors, respectively.

†† *R*_free_ was calculated using a randomly chosen 5% of reflections.

**Table 2:** Nucleotide-binding dynamics quantified by ITC

	*K*_d_ (µ*M*)	Δ*H* (kcal mol^−1^)	Δ*S* (cal mol K^−1^)	Δ*G* (kcal mol^−1^)
lpWalK (370–624)				
AMPPNP	450.50 ± 6.36	−2.76 ± 0.60	6.06 ± 0.02	−4.57 ± 0.60
ADP	1195.00 ± 16.97	−13.59 ± 0.64	−32.36 ± 2.94	−3.95 ± 0.64
AMP	1278.50 ± 95.46	−6.50 ± 1.31	−8.50 ± 2.08	−3.97 ± 1.32
smVicK (196–450)				
AMPPNP	538.50 ± 31.96	−9.50 ± 0.35	−16.88 ± 4.22	−4.47 ± 0.35
ADP	701.33 ± 165.17	−15.46 ± 0.87	−43.37 ± 36.96	−0.97 ± 0.87
AMP	2821.75 ± 370.47	−14.77 ± 0.64	−22.88 ± 11.31	−3.62 ± 0.64
smVicK CA (271–450)				
AMPPNP	292.00 ± 15.21	−11.31 ± 0.25	−21.72 ± 1.80	−4.83 ± 0.25
ADP	474.00 ± 97.49	−19.87 ± 0.50	−41.13 ± 10.75	−4.61 ± 0.50
AMP	852.67 ± 120.79	−10.35 ± 0.32	−17.97 ± 6.39	−4.30 ± 0.32
