# Supplementary material for: Conformational dynamics of the essential sensor histidine kinase WalK
Source: Acta Crystallogr D Struct Biol. 2017 Sep 27;73(Pt 10):793–803. doi: 10.1107/S2059798317013043 (PMC5633905; doi:10.1107/S2059798317013043)
Supplement: Supplementary file 6 [file d-73-00793-sup1.pdf]

STRUCTURAL BIOLOGY

Volume 73 (2017)

Supporting information for article:

Conformational dynamics of the essential sensor histidine kinase WalK

Yongfei Cai, Mingyang Su, Ashfaq Ahmad, Xiaojie Hu, Jiayan Sang, Lingyuan Kong, Xingqiang Chen, Chen Wang, Jianwei Shuai and Aidong Han

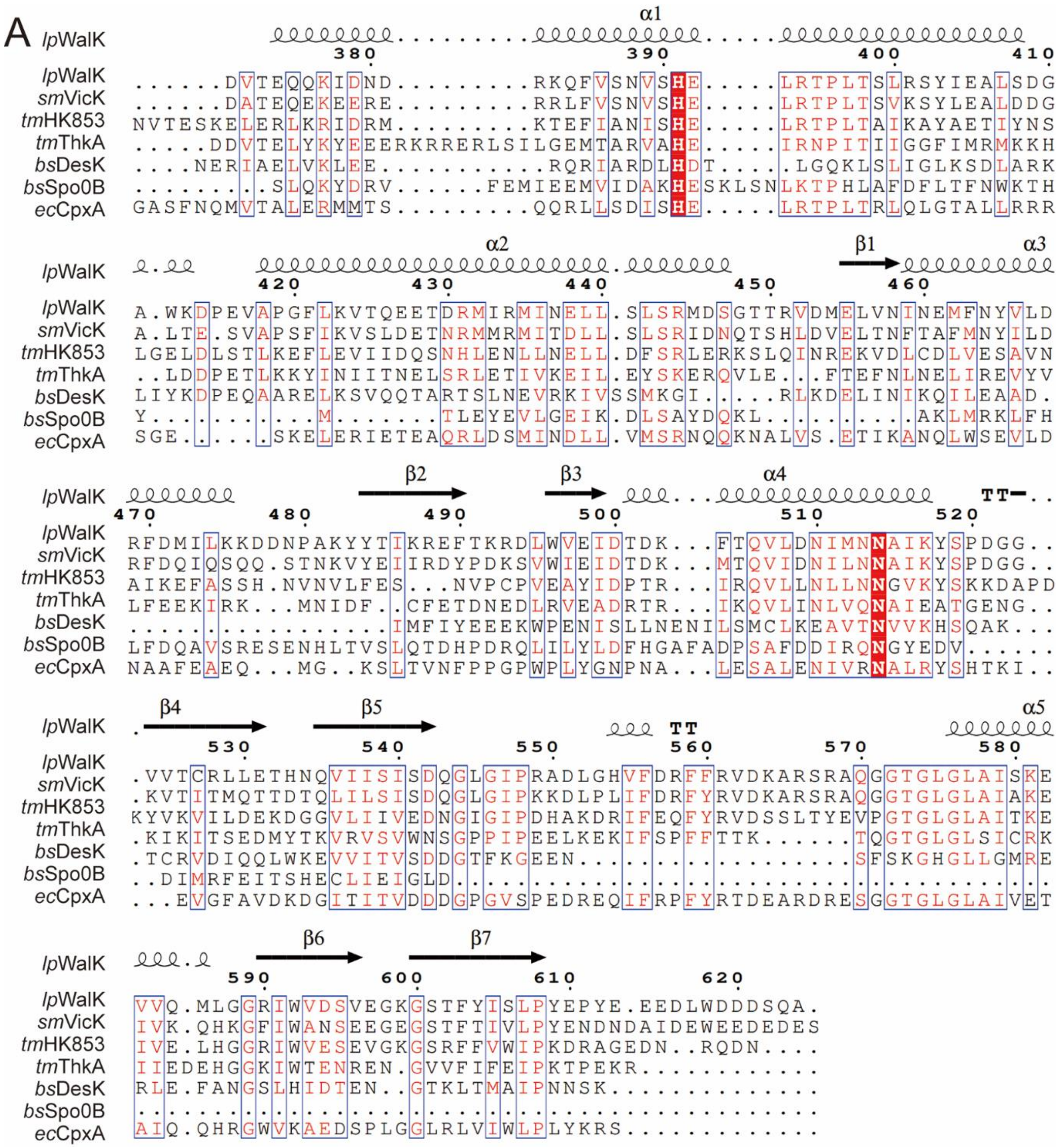

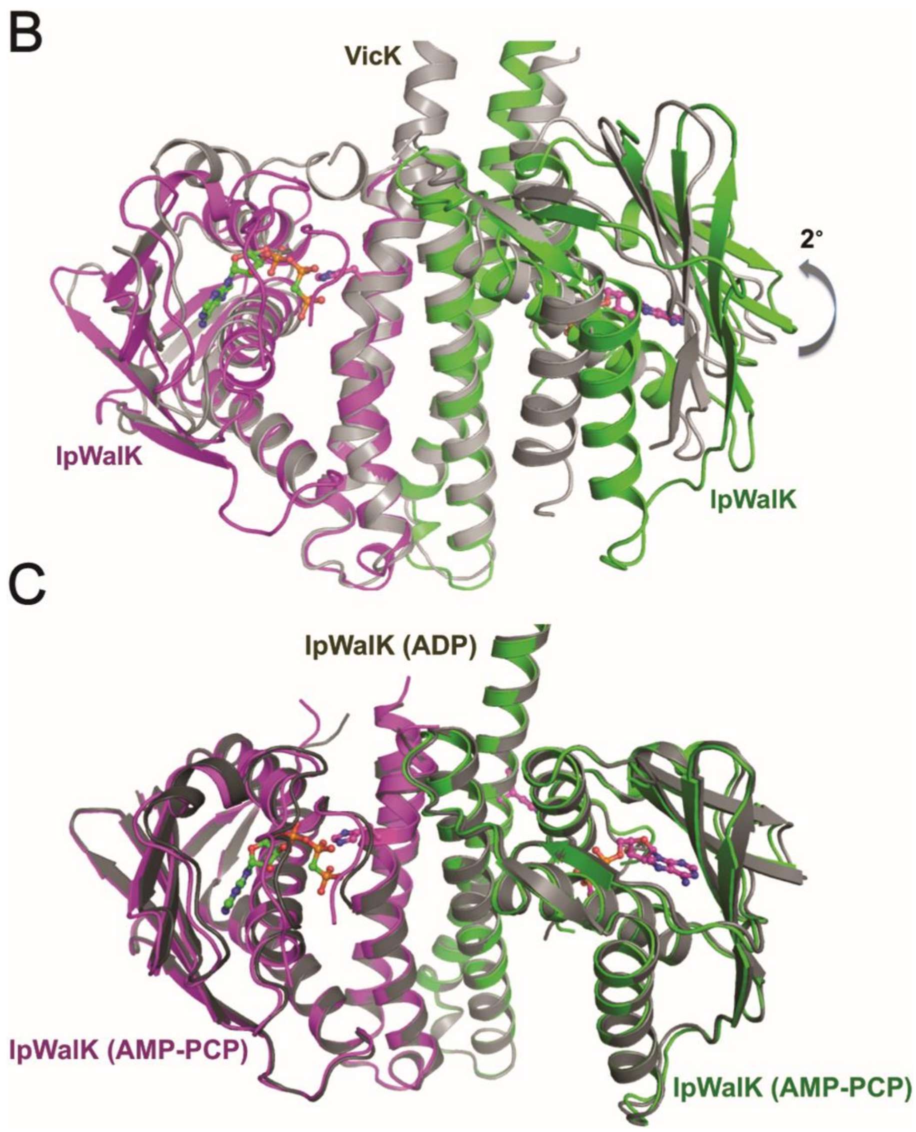

Figure S1 Global alignments of the lpWalK. (A) Sequence alignment of lpWalK with other SKs. The secondary structure elements are shown above the alignments as spiral lines and arrows for α helices and β strands. Highly conserved residues are shown in white letters over red background. Partially conserved residues are shown in red letters in blue boxes. These bacteria are *Lactobacillus plantarum*, *Streptococcus mutans*, *Thermotoga maritima*, *Bacillus subtilis*, *Escherichia coli*. (B) LpWalK aligned with the corresponding region of *S. mutans* VicK (smVicK). LpWalK is colored in magenta and green, and VicK in grey. The CA domain of the open chain of smVicK (rmsd of 1.1 Å for 186 Cα atoms) rotates 2° relative to the DHp, while a closed chain is well aligned for both domains (rmsd of 1.5 Å for 163 Cα atoms). The rotation angle of the open chains is labeled with a grey arrow on the right. (C) An alignment of lpWalK bound with AMPPCP (colored in magenta and green) with lpWalK bound with ADP (ADP is

not shown) at rmsd of 0.69 Å for 410 Cα atoms. The AMPPCP ligands bound in lpWalK are shown in sticks.

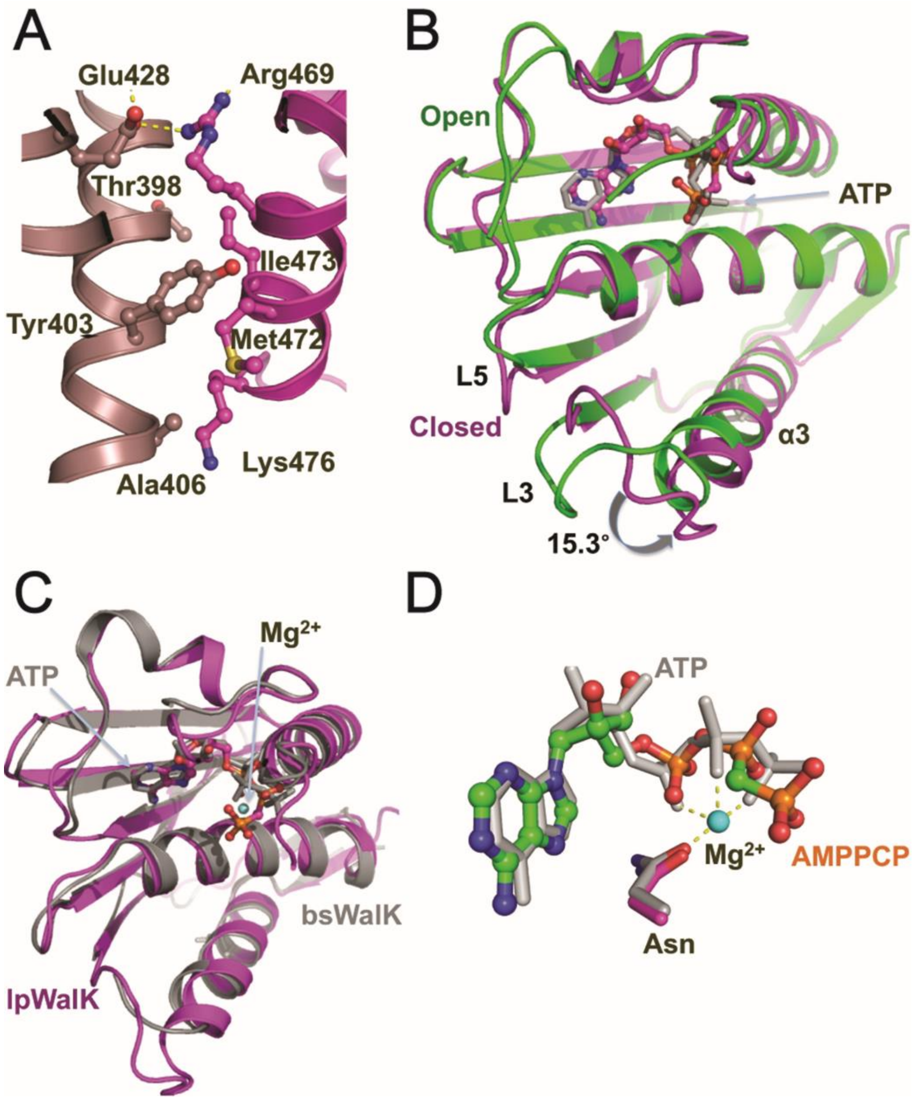

Figure S2 Detailed conformational analyses of the closed lpWalK state in presence of AMPPCP. (A)The hydrophobic patch between the CA and DHp domains in the chain with the closed conformation. The key residues are shown in sticks. A hydrogen bond is shown in yellow dashed line. (B) Structural rigidity of the CA domain of WalK. Alignment of the CA domains of lpWalK in the closed conformation with that in the open conformation. The CA domain of the closed conformation is colored with magenta, and the open conformation in green. A rotation angle of the helix α3 is indicated with a grey arrow. (C) Alignment of the lpWalK CA domain in the closed conformation with bsWalK. LpWalK is colored in

magenta, and bsWalK in grey. ATP in the closed chain of lpWalK is shown in orange sticks. (D) The AMPPCP conformation in lpWalK loses two coordinates with Mg^{2+}. ATP in bsWalK ia aligned to AMPPCP, showing coordinates of Mg^{2+}. Asparagines from both structures are shown in sticks. ATP in bsWalK is shown in grey sticks. The Mg^{2+} associated with ATP is shown in a cyan ball.

A. smVicK 196-450 and AMPPNP

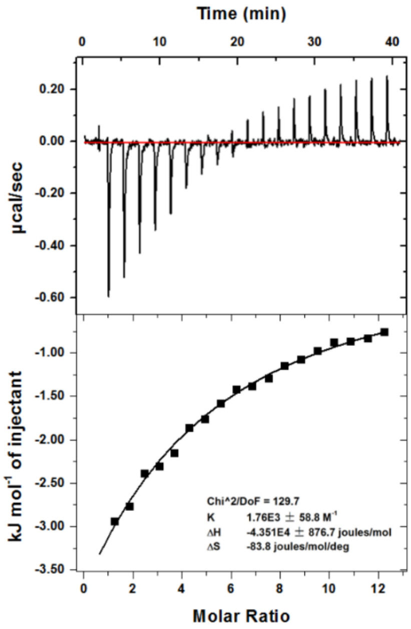

B. smVicK 196-450 and ADP

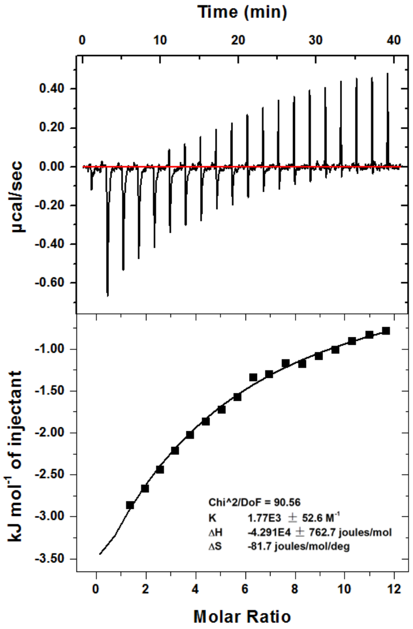

C. smVicK 196-450 and

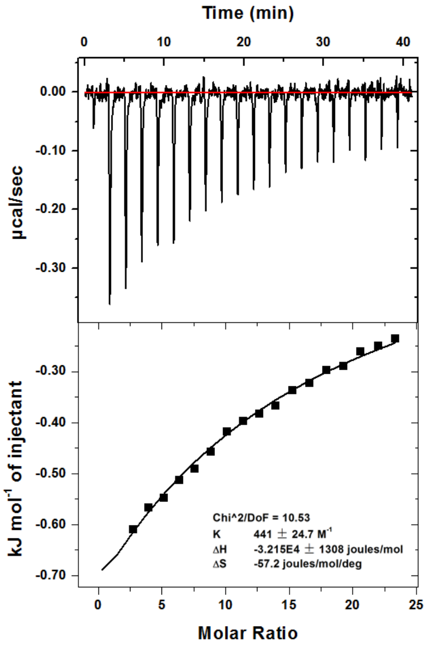

D. smVicK 271-450 and AMPPNP

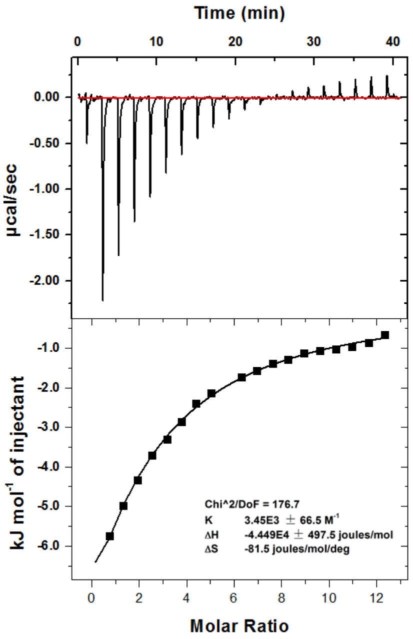

E. smVicK 271-450 and ADP

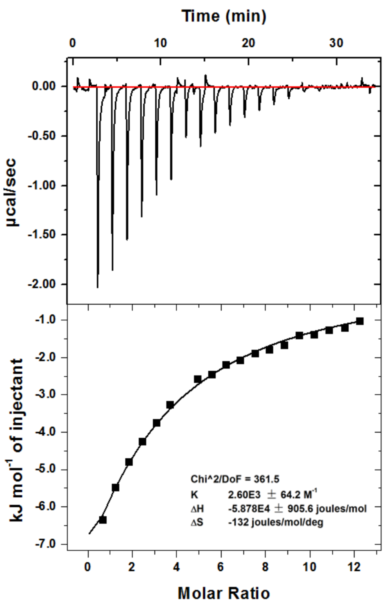

F. smVicK 271-450 and AMP

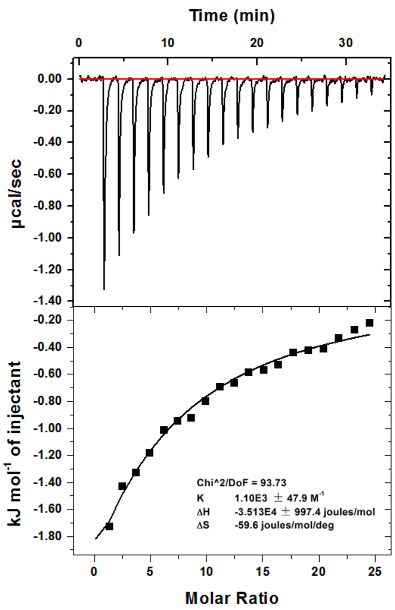

G. lpWalK and AMPPNP

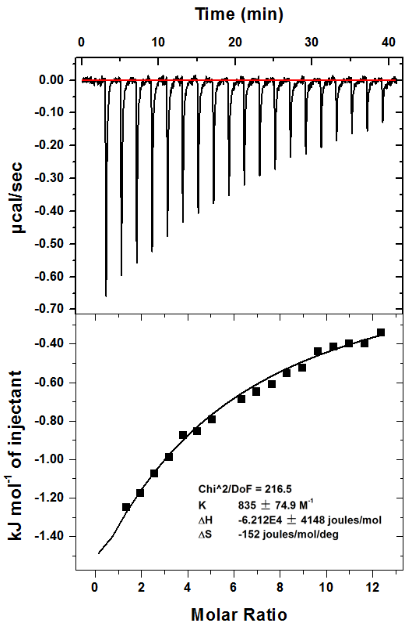

H. lpWalK and ADP

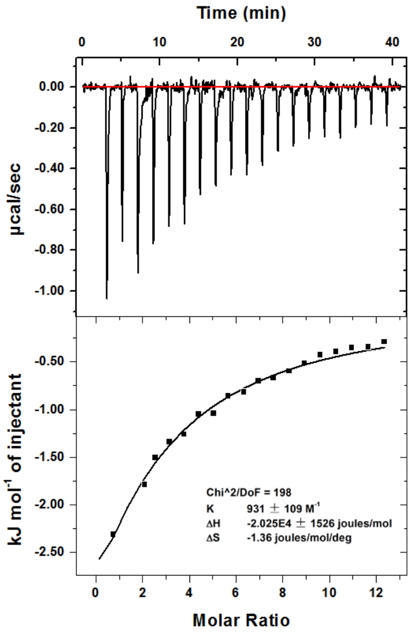

I. lpWalK and AMP

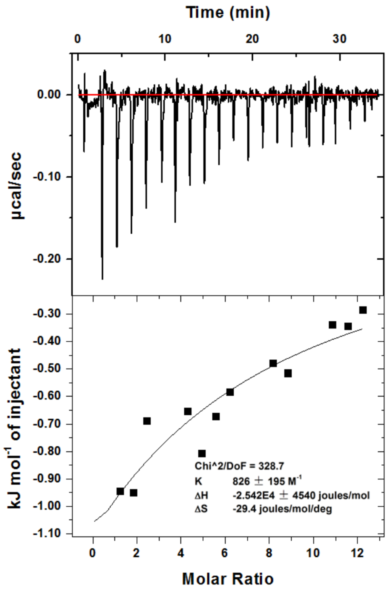

Figure S3 Quantifications for interactions of smVicK and nucleotides by Isothermal titration calorimetry. (A-C) The catalytic region of smVicK (aa 196-450) that comprises both DHp and CA domains was titrated with 3 mM AMPPNP, 3 mM ADP and 6 mM AMP, respectively. (D-F) The CA domain of smVicK (aa 271-450) was titrated with 3 mM AMPPNP, 3 mM ADP and 6 mM AMP, respectively. Nucleotides as injectants were simply diluted into the same buffer of 20 mM Tris-HCl, pH 8.0 and 5 mM $MgCl_2$. The titrations were performed at room temperature. (G-I) Titration of lpWalK (aa 370-624) with 3 mM AMPPNP, 3 mM ADP and 6 mM AMP, respectively. Nucleotides as injectants were diluted into the same buffer. The titrations were performed at room temperature.

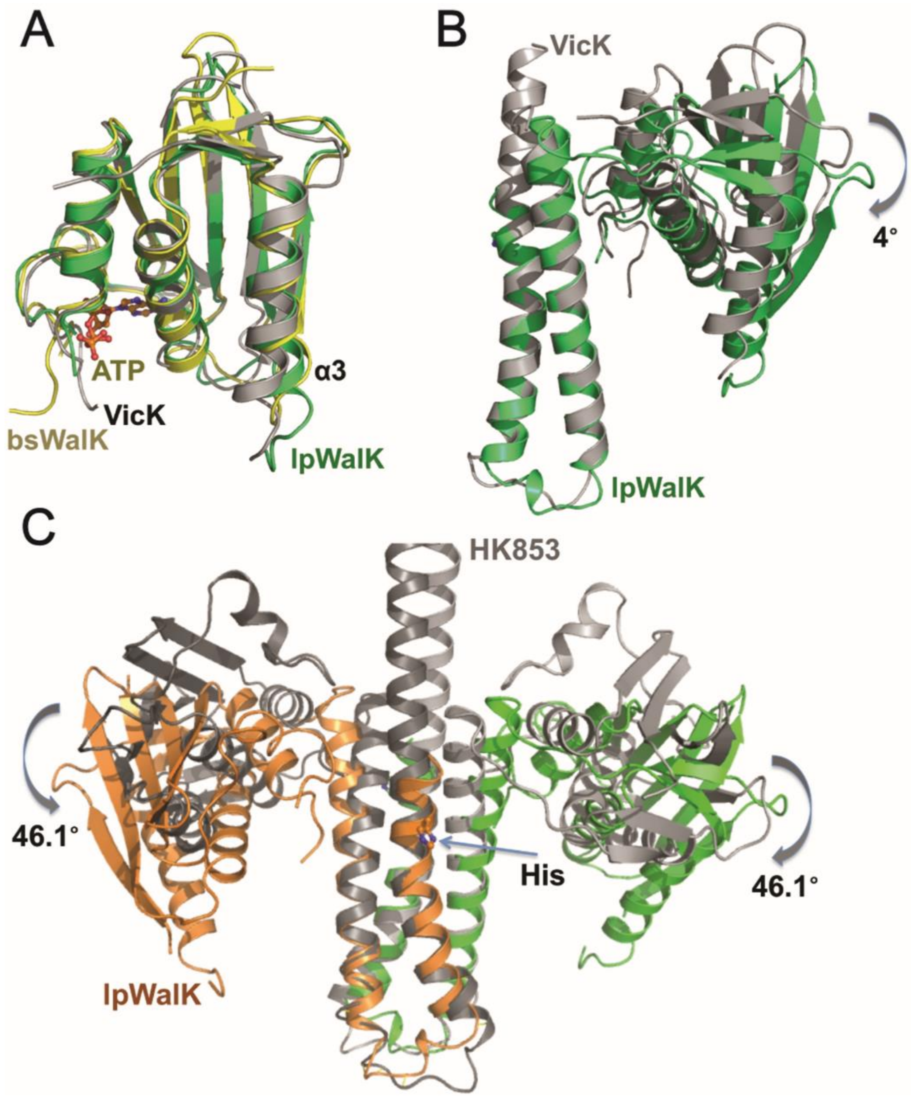

Figure S4 Comparisons of the open conformation of WalK. (A) The CA domain of lpWalK aligned with its homologs. The CA domain of *S. mutans* VicK is colored in grey, and lpWalK in green, and bsWalK in yellow. ATP from the bsWalK structure is shown in sticks. (B) The monomer of lpWalK aligned with the inactive chain of smVicK. (C) Global alignment of lpWalK with *T. maritima* HK853. LpWalK is colored in gold and green, and HK853 in grey. The phosphorylatable histidine is shown in sticks.

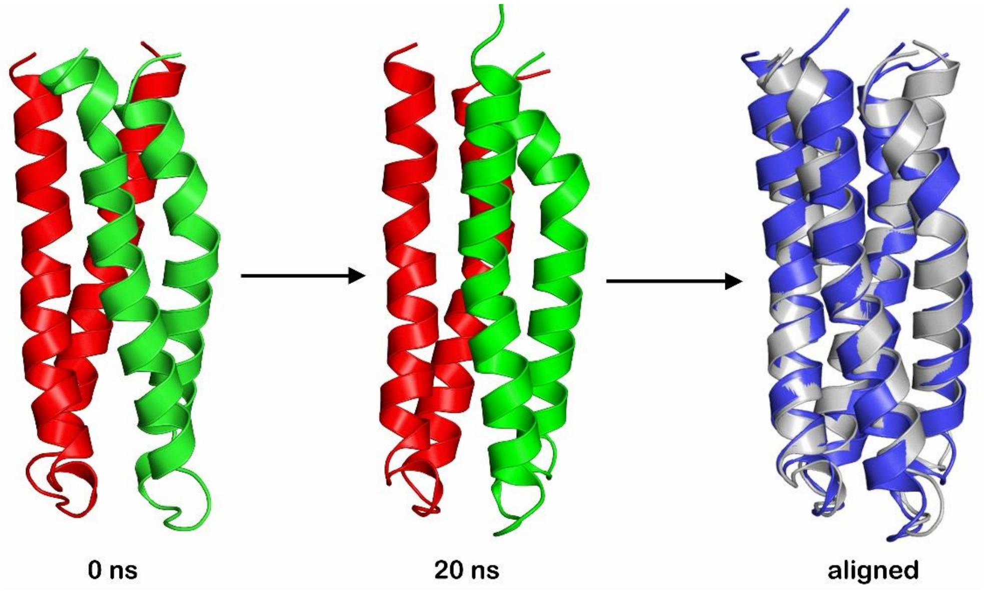

Figure S5 Relative stability of the DHp domain with the equal length of the helix α1 in molecular dynamic simulation for 20 ns. The simulated DHp domain (20 ns) in blue was aligned to its original conformation (0 ns) in grey on the right. No bending switch was observed.

Supplementary video S1. Conformational transition of lpWalK from the open state to the closed state. The homodimer is colored in magenta and green. The alignment based on the DHp domain is performed consistent to the Fig. 4.

Supplementary video S2. The molecular dynamic s. imulation of the DHp domain from the open state of lpWalK. The homodimer is colored in yellow and green. Pro396 as a bending point is highlighted with a red ball.

Supplementary video S3. The molecular dynamic simulation of the DHp domain from the closed state of lpWalK. The homodimer is colored in magenta and green. Pro396 as bending point is highlighted with a white ball.

Supplementary video S4. The molecular dynamic simulation of the engineered DHp domain from the closed state. The color scheme is the same as video 3. A short green helix on the top of magenta monomer is a helical extension switched from its green monomer.

Supplementary video S5. The molecular dynamic simulation of the engineered DHp domain from the closed state. The color scheme is the same as video 3. The extended helix of the green monomer was manually rebuilt to be a random coil.
